# Paced or Non-Paced? Examining an Electrocardiogram Rythm

**DOI:** 10.19102/icrm.2017.080602

**Published:** 2017-06-15

**Authors:** Martha Ferrara

**Affiliations:** ^1^Pacemaker Center, The Valley Health System, Ridgewood, NJ

**Keywords:** His-bundle pacing, electrocardiogram, right ventricular apex pacing

## Abstract

Many patients who present for pacemaker follow-up one week after device implantation have a “different” presenting rhythm, which can be puzzling to examine for even the most experienced cardiac device clinicians. Novel implanting techniques on the horizon necessitate a thorough understanding of these new technologies to provide safe care in this patient population.

## Case presentation

An active 79-year-old male with a history of fatigue and progressive shortness of breath with mild activity was seen in consultation due to his abnormal electrocardiogram (ECG), displaying progressive first-degree atrioventricular (AV) block prolongation over a period of years. An echocardiogram at the current visit revealed an ejection fraction of 55% to 65% with no valvular disease. The patient’s history includes hypertension, coronary artery disease, prior inferior wall myocardial infarction, biopsy-proven senile cardiac amyloidosis, and a six-month history of diastolic heart failure. The following question was raised: is this a pacemaker/paced rhythm?

## Discussion

**Figure 1:**
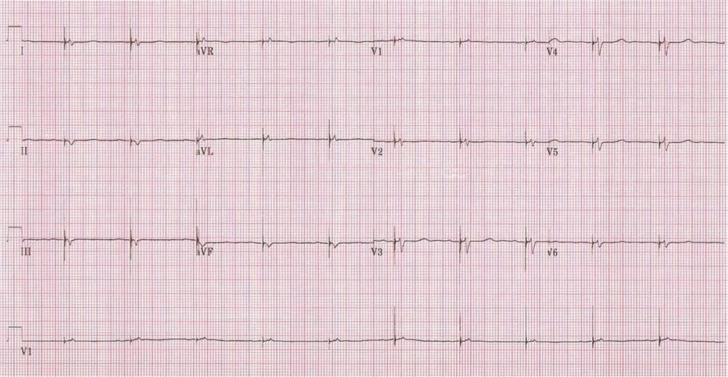
The patient’s ECG one week after pacemaker implantation.

**Figure 2:**
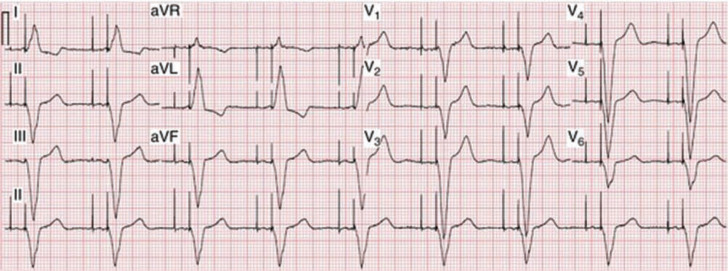
A dual-chamber pacing ECG.

Following additional examination, it was determined that this patient’s rhythm was a ventricular paced rhythm **(Figure 1);** however, the pacemaker lead was not located in the typical right ventricle (RV) apex (RVA), which produces the typical left bundle branch block pattern on an ECG **(Figure 2)**. Studies have shown that RVA pacing is associated with increased morbidity, mortality, heart failure and the development of persistent atrial fibrillation in comparison with native AV conduction.^1–3^

**Figure 3:**
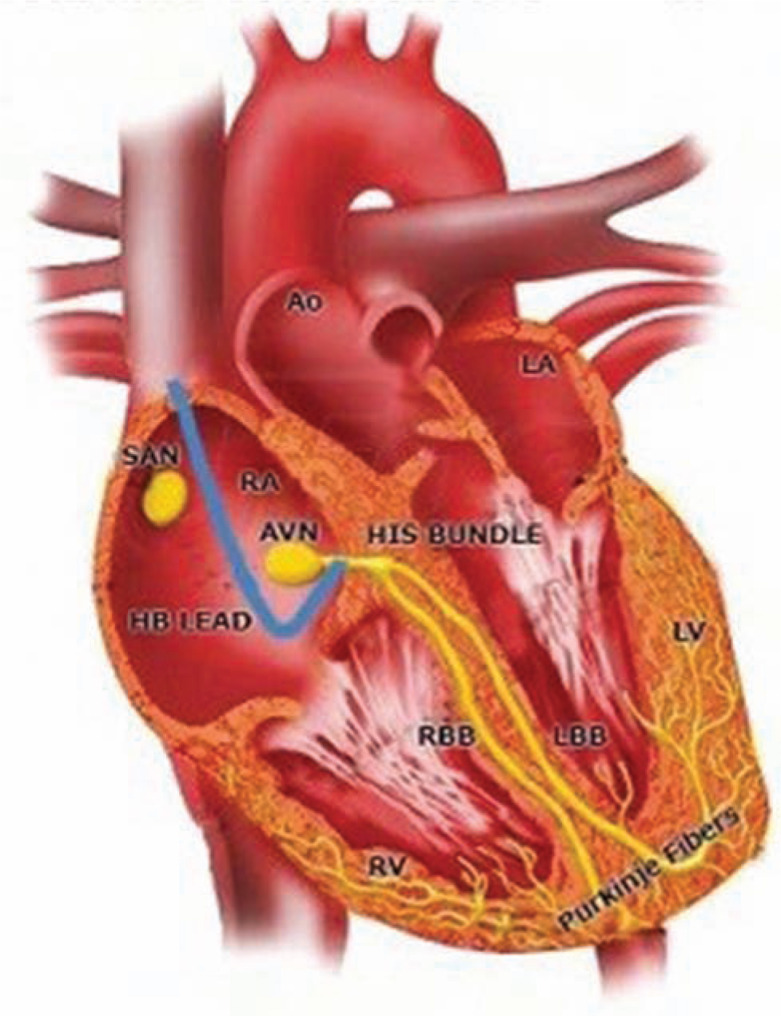
The cardiac conduction system. Public Domain Heart Conduction Image. Retrieved 2/12/2017 from https://s-media-cacheak0.pinimg.com/originals/22/de/be/22debe23d28fc684464fca3b31acdb2d.jpg.

**Figure 4:**
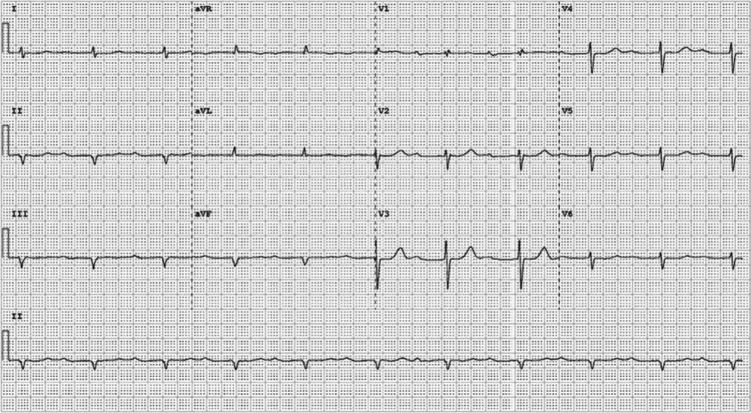
The patient’s ECG before the His-bundle pacemaker implantation.

**Figure 5:**
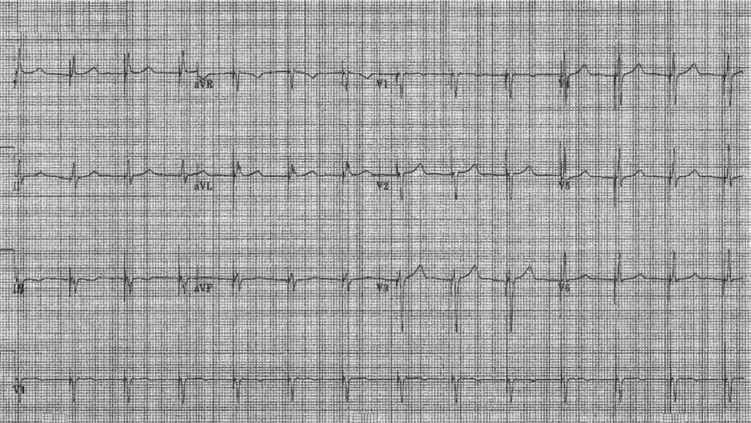
A para-Hisian His-bundle pacing ECG.

One of the goals of modern pacemaker technology, aside from its primary aim of maintaining a normal heart rate, is to mimic native conduction as closely as possible, thus avoiding a loss of synchrony and any potential long-term ventricular structural and/or functional changes. At the 36th Heart Rhythm Society Annual Scientific session, multiple abstracts were presented illuminating the “new” pacing method of His-bundle region pacing, though the concept has in fact been around for decades.^4–7^ This is an exciting and promising novel technique. In pacing the His-bundle area, ventricular activation follows the pattern of the intrinsic, natural electrical wavefront, depolarizing the ventricles, thus obviating the negative effects of RV pacing.^8^ The lead is generally placed above the tricuspid valve on the His bundle or Hisian region **(Figure 3)**, or as close to this region as possible (para-Hisian) and above the bundle branches, eliciting and promoting “normal” ventricular activation; hence, a narrow QRS is then seen on the ECG, as is presented in this patient’s presenting paced-ECG shown in **Figure 1.** The current patient’s “natural” or non-paced ECG present prior to the His-bundle pacemaker implant can be seen in **Figure 4,** with the note that the width of QRS is very similar to the paced QRS post-His-bundle pacemaker implantation. If His-bundle lead placement or capture is not feasible, a para-Hisian location may be acceptable as noted by the ECG demonstrated in **Figure 5,** which denotes no isoelectric line between the pacemaker spike and the slurring deflection capturing the His-bundle and local ventricular tissue. Of note, the R-wave and T-wave complexes are concordant (ie, both upright) as is seen in the patient’s natural ECG.

Technical difficulties in lead positioning, a lack of appropriate tools, limited clinical data, and the novelty of the procedure have been cited as some of the barriers to the widespread use of this approach by physicians implanting pacemakers.^4,9^ His-bundle pacemaker SelectSecure pacing leads (model 3830; Medtronic, Minneapolis, MN, USA) and a fixed-shape catheter (C-315; Medtronic, Minneapolis, MN, USA) used to deliver the pacing lead to the His-bundle region are currently the only such tools on the market relevant for use during this procedure. Up-to-date guidelines for pacemaker implants are followed, with patients selected for His-bundle pacing procedures based on their adherence to the criteria: for example, patients with advanced infiltrative or infra-Hisian disease may not qualify for this type of pacing lead. During implantation, the electrophysiologist can make this assessment, as was demonstrated by Sharma et al.^8^ in a study that demonstrated the lead’s safety and feasibility without necessity of RV back-up leads or His-bundle mapping catheters. However, new studies also show that careful consideration should still be given to patient selection as is the case with the use of any novel technology.^10^

**Figure 6:**
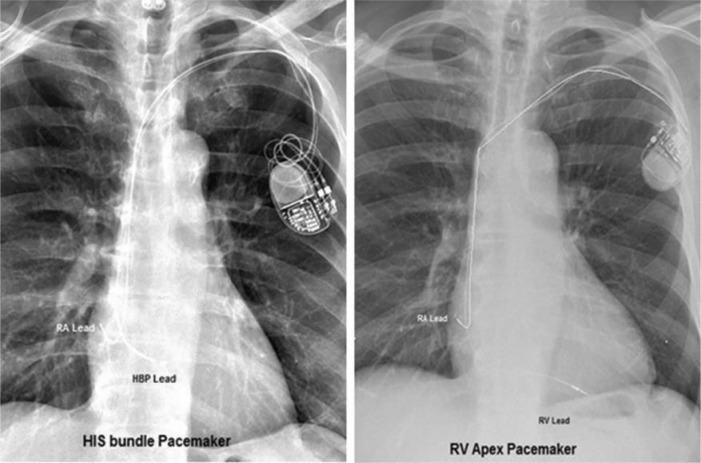
Locations of the His-bundle pacemaker (left) and RV apex pacemaker (right).

Patients presenting to the device clinic for the first follow-up, typically one week post implant for a wound check, have a “different” presenting rhythm. At first, it appears as though no ventricular pacing is present due to the “natural” R-wave look (narrow), which closely mimics the patient’s intrinsic R-wave, especially if the lead is located at the His-bundle (Hisian) region. As such, the device clinician must be aware of the type of pacemaker and lead(s) implanted. The operative report is almost always helpful for confirming device implant technique and location of lead placement. A chest X-ray is also helpful for identifying lead placement, as the ventricular lead shows in a different anatomic position than a typical RV apical lead **(Figure 6)**. During clinical follow-up, a 12-lead ECG performed during threshold measurement can help identify His-bundle and RV threshold capture, though this is normally not done during standard RV pacemaker follow-up evaluation. Device programming is also different from customary settings; notably, R-waves are usually lower in amplitude from the His-bundle region, and thus acceptable programming varies in ventricular sensitivity (less sensitive), threshold (slightly higher) and polarity (unipolar) from typical RVapex settings. The importance of educating the heath-care team to novel technologies in order to deliver appropriate care cannot be overstated.
